# Effectiveness of Non-Pharmacological Interventions in Patients with Facial Paralysis: An Umbrella and Mapping Review

**DOI:** 10.3390/medicina61081502

**Published:** 2025-08-21

**Authors:** Mónica Grande-Alonso, Alba Ayllón-Poza, Álvaro Saavedra-Böss, Nayra Daniela Santa Cruz-Saavedra, Celia Vidal-Quevedo, Carlos Forner-Álvarez, Ferran Cuenca-Martínez

**Affiliations:** 1 Departamento de Cirugía, Ciencias Médicas y Sociales, Facultad de Medicina, Universidad de Alcalá, 28871 Alcalá de Henares, Spain; 2 Grupo de Investigación Clínico-Docente Sobre Ciencias de la Rehabilitación (INDOCLIN), Centro Superior de Estudios Universitarios La Salle, 28023 Madrid, Spain; 3 Centro Superior de Estudios Universitarios (CSEU) La Salle, Universidad Autónoma de Madrid, 28023 Madrid, Spain; 4 Servicio de Rehabilitación, Instituto de Investigación Sanitaria Fundación Jiménez Diaz (IIS-FJD, UAM), Hospital Universitario Rey Juan Carlos, 28040 Madrid, Spain; 5 Faculty of Physiotherapy, University of Valencia, c/Gascó Oliag n°5, 46010 Valencia, Spain; 6 Department of Physiotherapy, University of Valencia, c/Gascó Oliag n°5, 46010 Valencia, Spain

**Keywords:** facial paralysis, non-pharmacological interventions, umbrella review

## Abstract

*Background and Objectives*: Facial paralysis involves the complete or partial loss of facial movement due to damage to the facial nerve, leading to impaired voluntary muscle function and facial asymmetry. Given its significant physical and psychosocial impact, there is an urgent need to strengthen the evidence supporting non-pharmacological treatments. This umbrella review aims to compile the most reliable and current data to establish a consensus on the effectiveness of such interventions for patients with facial paralysis. *Materials and Methods*: This study is an umbrella review. A systematic search was conducted in PubMed, Embase, Scopus, and CINAHL (28 July 2024). The variables assessed included overall healing/recovery rate, facial disability, and facial function. Methodological quality was evaluated using the AMSTAR and ROBIS tools. Screening was performed independently by two reviewers, with a third reviewer resolving any discrepancies. *Results*: Five systematic reviews were included, all evaluating the impact of non-pharmacological interventions in facial paralysis. The findings suggest that acupuncture and electrical stimulation may improve recovery rates and facial function, although high heterogeneity and methodological limitations were noted in some studies. No definitive conclusions could be drawn regarding facial disability. *Conclusions*: The combination of electrotherapy with other complementary techniques, such as facial exercises or laser therapy, appears to be a safe and potentially effective approach for facial paralysis rehabilitation. Nonetheless, further research employing standardized protocols and higher methodological quality is necessary to establish more robust conclusions for physiotherapeutic practice.

## 1. Introduction

Facial paralysis (FP) is characterized by the complete or partial loss of facial movement resulting from damage to the facial nerve. This condition disrupts the voluntary motor function of the facial muscles, leading to facial asymmetry [1].

FP is classified based on the lesion’s location: peripheral facial paralysis (PFP), which involves the lower or peripheral motor neuron, and central facial paralysis (CFP), which results from a lesion in the central or upper motor neuron [2]. PFP originates from nerve damage at the brainstem level, affecting the facial muscles on the lower, middle, and upper regions of one side of the face [2]. Causes of PFP may include traumatic events such as skull fractures, as well as infectious, neoplastic, or idiopathic factors, with Bell’s palsy (BP) being the most common cause within this category [3,4]. Conversely, CFP occurs due to neurological impairment in the motor cortical areas, often caused by stroke, and typically affects only the lower half of one side of the face [2].

As previously mentioned, idiopathic facial paralysis, or Bell’s palsy, is the most frequent cause of PFP, with an incidence of 20–35 cases per 100,000 individuals annually, accounting for 60–75% of unilateral facial paralysis cases [5,6]. The prevalence of this condition is similar across genders, with the highest incidence observed in individuals aged 40 to 49 years [7]. The rate of complete recovery from FP ranges between 70 and 75%, although 12% of patients may experience mild residual weakness, 13% moderate weakness, and 4% severe weakness [8]. Complications can include muscle tension in 17% of cases and the development of synkinesis or blepharospasm in 16% [9].

Key symptoms associated with FP include the inability to blink, raise the corner of the mouth, or purse the lips. Additional signs may include drooping of half the eyebrow and/or face, ear pain, flattening of the nasolabial fold, dehydration of the eyes or mouth, and hearing loss. In some cases, hyperacusis and paresthesia may also be present on the affected side [10,11].

There are variations in symptom presentation depending on the type of paralysis. CFP results in impairment of the lower quadrant of the face on the side opposite the lesion, without involvement of the periocular muscles, whereas PFP affects all facial muscles, including the fronto-lateral musculature [12]. The onset of symptoms is generally sudden, and the severity can range from mild fatigue to severe paralysis [13]. Typically, within 24 h, patients experience acute motor deficits in the ipsilateral muscles, with the most severe symptoms usually peaking within the first 72 h [14]. Facial paralysis can lead to muscle hyperactivity both at rest and during voluntary movement, significantly impacting the patient’s quality of life, both functionally and psychosocially [14,15]. In addition to its sensorimotor effects, FP can have psychological consequences, contributing to anxiety, depression, and low self-esteem, with social disability often being more impactful than the functional deficits themselves [15,16,17]. Given the central role of the face in identity and social interaction, it is crucial to address these psychosocial factors in FP management to optimize recovery [17,18]. Regarding the progression of FP, approximately 13% of patients experience mild paresis, 4–5% retain significant facial dysfunction, while the majority achieve full recovery [19]. Several therapeutic interventions are available to alleviate symptoms and promote rehabilitation [10]. These include corticosteroids, such as prednisone, to reduce inflammation; surgical procedures like nerve grafts and muscle transfers; as well as ocular care, occupational therapy, and speech therapy to improve swallowing and speech [10,20,21,22]. Although pharmacological treatment remains the most commonly used approach, recent research highlights the importance of non-pharmacological therapies, which have shown notable benefits. These include electrical stimulation (ES) [23,24,25], mimic therapy [23,26,27], biofeedback [23,26,27,28], proprioceptive neuromuscular facilitation [23,27,29], massage therapy [26,30,31], facial exercises [26,27,28,32,33], and mirror therapy [29,32,34]. These methods can be administered individually or combined with other interventions to enhance recovery. Moreover, evidence suggests that early intervention is critical in preventing maladaptive behaviors that could hinder the rehabilitation process [35].

To date, no umbrella review has synthesized the evidence across various non-pharmacological therapies for FP; because of that, the aim of this umbrella review is to compile the most credible and up-to-date information in order to establish a consensus regarding the efficacy of non-pharmacological interventions in patients with FP.

## 2. Materials and Methods

### 2.1. Protocol and Registration

A protocol was developed in accordance with the Preferred Reporting Items for Systematic Reviews and Meta-Analysis Protocols (PRISMA-P) [36] and was registered in the Prospective Registry of Systematic Reviews (PROSPERO) [37]. This registration is publicly available under the number CRD42024620207. Furthermore, the reporting of this study adheres to the Preferred Reporting Items for Overview of Systematic Reviews Checklist (PRIO-harms) [38].

### 2.2. Search Strategy

On the 28 July 2024, we conducted a search for published scientific articles in the following databases: PubMed, Embase, Scopus, and CINAHL. The search was performed by two independent reviewers who employed the same methodology, and any discrepancies arising during this phase were resolved through consensus. Additionally, the reference lists of the included and original studies were manually screened. Supplementary Annex S1 shows the search strategies used for each database.

### 2.3. Eligibility Criteria and Data Extraction

The eligibility criteria employed in this article were based on the PICO format, written as follows:

− Population: patients with facial paralysis.
− Intervention: any non-pharmacological treatment that is the primary intervention and that is applied in isolation or together with additional treatments.
− Comparison: any treatment other than the primary intervention.
− Outcomes: (including at least one of the following): cure/full recovery rate, facial disability, or facial function.

At first, two independent reviewers performed a screening to assess the relevance of the systematic reviews (with or without meta-analysis) in relation to the research questions and objectives of the studies. The initial screening was based on the title and abstract of each paper. If consensus could not be reached or if the abstracts were deemed insufficient, the full text was reviewed. In the second phase of the screening, the full text was evaluated to determine whether the studies met all inclusion criteria. Discrepancies between the reviewers were resolved through discussion and a consensus process, facilitated by a third reviewer. Lastly, the data extraction was performed using a structured protocol, ensuring that only the most relevant information was obtained from each study (population characteristics, study quality and risk of bias, outcomes, interventions and their main parameters, and number of studies included).

### 2.4. Methodological Quality Assessment

Two independent reviewers evaluated the methodological quality of the systematic reviews included in the study. Additionally, any disagreements in the final quality assessment score were resolved through consensus with a third independent reviewer. The reviewers used the Modified Quality Assessment Scale for Systematic Reviews (AMSTAR) [39], which consists of 13 items, each worth 2 points (with “yes” scoring 2, “in part” scoring 1, and “no” scoring 0), with a maximum possible score of 26. Furthermore, the AMSTAR is a tool that has been proven to be both valid and reliable for assessing the methodological quality of systematic reviews. A cut-off score of 20 or more points, indicating high quality.

### 2.5. Risk of Bias Assessment

The risk of bias of the systematic reviews included in the study was evaluated by two independent reviewers using the Risk of Bias in Systematic Reviews (ROBIS) tool. Any discrepancies in the final quality assessment score were resolved through consensus with a third independent reviewer. The ROBIS tool is composed of three phases and includes a series of questions that are answered with “yes”, “probably yes”, “probably no”, “no”, or “no information”. Based on the responses provided, the risk of bias is subsequently classified as “low”, “unclear”, or “high” [40].

### 2.6. Evidence Map

A visual representation of each systematic review was created to effectively present the information. The evidence map is based on four distinct dimensions:

− Strength of findings (*y*-axis): AMSTAR
− Effect size (*x*-axis): The authors categorized each systematic review based on the effects observed. If the primary intervention demonstrated greater benefits than the comparator, the intervention was classified as “potentially better.” Conversely, if the comparator showed greater benefits, the intervention was classified as “potentially worse.” If the results were contradictory, the intervention was labelled as having “mixed results.” If no differences were observed, the intervention was classified as showing “no differences.” In cases where the evidence was insufficient, the intervention was categorized as “unclear.”
− Outcomes measured (triangle color): cure/full recovery rate (dark blue), facial disability (pink), and facial function (purple).
− Number of studies (triangle size): The size of each triangle is proportional to the number of primary studies included in each systematic review.

Finally, it should be noted that this evidence map is an original creation, although it was inspired by the one used by Fuentes-Aparicio et al. [41].

## 3. Results

### 3.1. Study Selection

**Figure 1:**
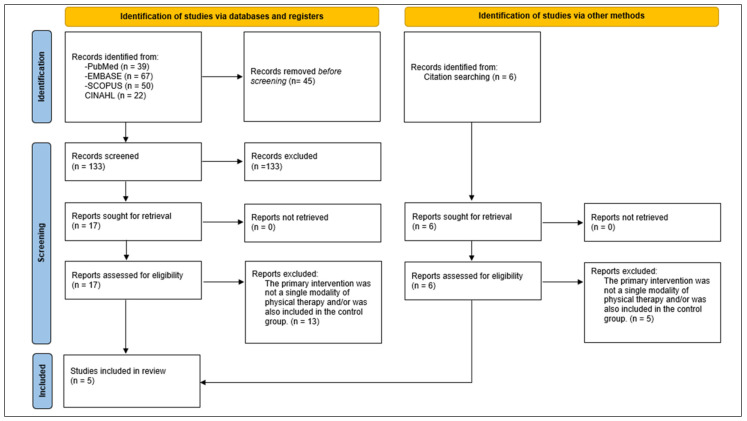
PRISMA Flowchart of studies selection.

The initial search revealed 178 total results. After the title and abstract screening and the full-text assessment, five systematic reviews were selected according to our eligibility criteria. The study selection process is shown in Figure 1.

### 3.2. Characteristics of Included Studies

The included SRs were published between 2017 and 2024. Regarding the primary interventions, two SRs had acupuncture [42,43], one low-level laser therapy (LLT) [33], and two ESs [24,25]. As for primary articles, there was only a 15% overlap. Further details on the characteristics of SRs can be found in Table 1. In addition, Table 2 shows the prescription parameters of the different primary interventions used.

### 3.3. Results of the Methodological Quality Assessment

Table 3 shows the results of the methodological quality assessment using AMSTAR. Two of the five SRs were considered high quality (20 points or more) [25,43], with 14 being the minimum score and 23 being the maximum score.

### 3.4. Results of the Risk of Bias Assessment

The findings of the risk of bias assessment using ROBIS are presented in Table 4, which indicates that 40% of the included systematic reviews were classified as having a low risk of bias.

### 3.5. Outcomes Measured

#### 3.5.1. Cure/Full Recovery Rate

A total of four SRs evaluated the effects of a non-pharmacological intervention on the degree of complete recovery in patients with FP [24,25,42,43].

Burelo-Peregrino et al. [24] found that the overall recovery rate was 96% in the experimental group (ES) and 88% in the control group. Furthermore, Fargher & Coulson [25] observed that ES favored complete recovery at one-year follow-up. However, in both cases, due to the methodological heterogeneity of the included studies, the findings need to be viewed with caution.

On the other hand, Chen et al. [42] evaluated the results of six articles that include acupuncture as the main intervention. In the experimental groups, recovery rates were 52% (30 cases), 74% (48 cases), 83.3% (25 cases), 63%, 62.5%, and 23.3%. The recovery rates in the control groups were 12% (6 cases), 45% (30 cases), 45% (9 cases), 17%, 63%, and 13.3%, respectively. Significant differences favoring acupuncture were observed between experimental and control groups (*p* < 0.01) in four of the six studies. In addition, Zhang et al. [43] reported a total recovery rate of 59.7% with acupuncture compared to 32.5% with pharmacological treatment; acupuncture was associated with an increased healing rate (RR = 1.77, 95% CI: 1.41–2.21), although significant heterogeneity was present among the pooled results (I^2^ = 67%, *p* = 0.0008).

#### 3.5.2. Facial Disability

Two SRs assessed the effectiveness of a non-pharmacological intervention on the facial disability levels of patients with FP [24,33]. The facial disability was evaluated using mainly the facial disability index (FDI) [44].

On one hand, Javaherian et al. [33] found in two of the four articles, that there was a significant improvement in the functional disability index among patients with FP following the application of LLT. On the other hand, Burelo-Peregrino et al. [24] included among their results the potential increase in facial disability when applying ES.

It should be noted that in both SRs, due to the great heterogeneity and the low methodological quality of their procedures, it cannot be concluded that there were significant effects regarding an improvement in facial disability.

#### 3.5.3. Facial Function

Three SRs evaluated the effectiveness of a non-pharmacological intervention on the facial function levels of patients with FP [24,25,33]. The facial function was assessed using the House–Brackmann Grading System (HBGS). The HBGS assesses facial function using a scale from I to VI, where I indicates normal movement and VI signifies complete paralysis. [45].

Burelo-Peregrino et al. [24] found that ES produced positive effects on facial function in patients with PF across all evaluated studies. However, due to the difference in methodology between the included studies and the large heterogeneity, the results should be interpreted with caution. On the other hand, Fargher and Coulson [25] did not find sufficient evidence to support the use of ES in FP for improving facial function. Finally, Javaherian et al. [33] found mixed results in on the efficacy of LLT in facial function in patients with FP.

### 3.6. Evidence Map

**Figure 2:**
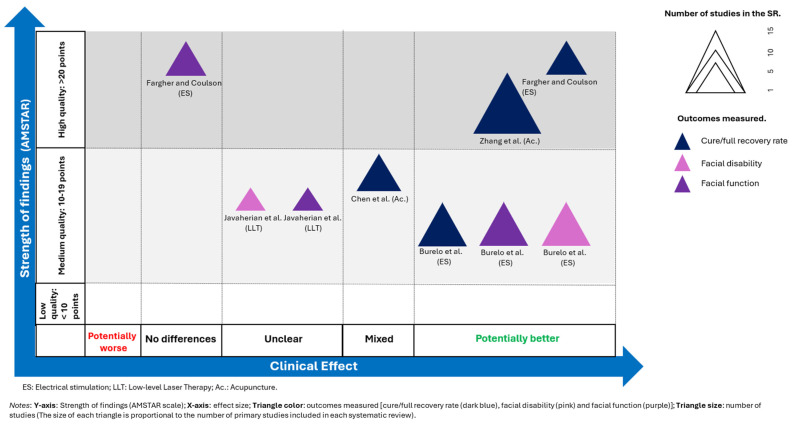
Evidence map [24,25,33,42,43].

The evidence map is shown in Figure 2.

## 4. Discussion

The primary aim of this umbrella review was to assess the effectiveness of non-pharmacological interventions on the recovery rate or complete healing, functional disability index, and facial function in individuals with facial paralysis.

Regarding the recovery rate in FP patients, our findings on electrical stimulation align with the existing literature. Specifically, adding selective electrical muscle stimulation to conventional physiotherapy—comprising exercises and massage—for acute Bell’s palsy has been linked to significantly faster recovery from flaccid paralysis, with comparable outcomes in terms of static facial expression, dynamic facial movement, and synkinesis [46].

Concerning acupuncture, results from three randomized controlled trials [47,48,49] are consistent with our findings, indicating that recovery and healing rates decreased when this therapy was applied.

In the trial conducted by Fahmy et al. [47], a significant reduction in HBGS scores was observed in both the acupuncture with moxibustion group and the pharmacological treatment group. Notably, the reduction was statistically greater in the acupuncture with moxibustion group compared to the pharmacological group at both three and six weeks post-treatment (*p* < 0.001). However, the study did not differentiate between the individual effects of acupuncture and moxibustion, making it unclear which component was responsible for the observed outcomes.

Martín Piñero et al. [49] reported that acupuncture alone achieved a 79% success rate, surpassing pharmacological treatment at 72%. Moreover, the combined therapy of acupuncture and medication yielded a higher recovery rate of 92%, suggesting greater effectiveness when both treatments are used together. These findings are similar to our results, although the studies included in our review evaluated acupuncture and pharmacological interventions separately rather than as a combined approach.

Regarding the FDI, our review identified significant improvements in FDI scores following the application of LLT, although these effects were not consistent across all included studies. These findings contrast with those of Kandakurti et al. [50], who reported sustained and clinically meaningful improvements in patients treated with LLT, especially when combined with facial expression exercises. This suggests that the effectiveness of laser therapy may depend on the mode of application and its integration with complementary therapies.

As for ES, Johannes et al. [51] documented substantial improvement in physical function—as measured by the FDI—following an individualized treatment protocol using electromyography-triggered stimulation in a clinical case of central facial paralysis. Conversely, Tuncay et al. [52], a primary article included in this umbrella, in an experimental study comparing conventional physiotherapy with a combined physiotherapy and ES approach, found that both groups showed favorable progress in FDI scores, but no significant differences were observed between them. This raises questions about the added value of ES within a combined treatment framework.

With regard to acupuncture, Teixeira et al. [30] noted that existing studies do not provide conclusive evidence about its impact on facial disability due to methodological limitations. While one reviewed study reported improved FDI scores, the lack of statistical robustness prevents these results from being considered definitive. Thus, acupuncture appears to be a potentially beneficial intervention, but current evidence remains insufficient to firmly support its efficacy in this area.

Regarding facial function in individuals with FP, our review identified positive outcomes following the use of ES and LLT. However, these results were not statistically significant when compared to control groups, and the overall quality of the available evidence was limited due to methodological shortcomings. Consistent with our findings, Lin et al. [53] reported in their meta-analysis significant improvements in facial function in FP patients treated with LLT, with no adverse effects observed. Moreover, their review concluded that LLT was more effective than ES, leading to a faster recovery of facial function in the LLT group. Notably, their study selection was based on high methodological quality, strengthening the reliability of their conclusions. Similarly, Macías-Hernández et al. [9] supported the safety and clinical benefits of LLT for improving facial function in FP patients. Along the same lines, one of the primary articles included in this umbrella, Alayat et al. [54], compared the effectiveness of LLT with high-intensity laser therapy (HILT), both of which led to significant improvements. However, their findings suggested that HILT was the more effective option. This insight highlights a promising avenue for future research focused on evaluating the efficacy of HILT compared to other therapies in patients with FP. However, several studies consistent with our findings have reported improvements in facial function using ES [46,52].

These findings suggest that both ES and LLT, when applied through various modalities, appear to be safe and effective non-pharmacological options for improving facial function in patients with facial paralysis. Nevertheless, further high-quality research is needed to strengthen the evidence base and confirm these outcomes.

### Limitations

This review presents several important limitations. First, there was significant methodological heterogeneity among the included reviews, which used various measurement scales—such as the HBGS and FDI—and different intervention protocols, making direct comparison of results across studies challenging. Second, the limited number of reviews included may also be considered a constraint. Thirdly, the overall scores obtained in both AMSTAR and ROBIS can also be considered a limitation. Another limitation is that data on some characteristics of the studies that may be of interest, such as type of FP, timing of intervention, or severity, were not extracted. Lastly, although the overall sample size was substantial and included patients of varying ages, the lack of access to the full texts of some primary studies may have hindered comprehensive data collection, potentially affecting relevant findings.

Therefore, further research is needed to develop standardized treatment guidelines based on more robust evidence and rigorous methodological designs to validate current findings.

## 5. Conclusions

Considering the methodological quality assessments (AMSTAR and ROBIS), which showed greater consistency in the studies on electrotherapy and laser light therapy, it can be inferred that combining electrotherapy with complementary approaches like facial exercises or laser therapy seems to offer an effective and safe rehabilitation strategy for facial paralysis. Nonetheless, several uncertainties persist, highlighting the need for future research to prioritize controlled clinical trials using standardized protocols. Such efforts are essential to resolve existing ambiguities and to establish clear, evidence-based guidelines for physiotherapy practices.

## Figures and Tables

**Table 1:** Characteristics of the reviews included in the umbrella review.

Author [Study Design] (No. of Studies Included)	Patients with Facial Palsy	Primary Intervention	Comparator	Outcomes Measured	Authors’ Conclusions
Burelo-Peregrino et al., 2020 [24] [SR] (7)	N = 131	ES	No ES treatment.	1-Facial function (HBGS). 2-Cure/full recovery rate. 3-Facial disability (FDI).	Electrical stimulation may improve facial muscle function.
Chen et al., 2010 [42] [SR] (6)	N = 537	Acupuncture	Placebo, usual care (drugs) or any other intervention without acupuncture.	1-Cure/full recovery rate.	Acupuncture may have a beneficial effect in treating FP.
Fargher and Coulson 2017 [25] [SR] (5)	N = 258	ES	Placebo, usual care (drugs) or any other intervention without electrical stimulation.	1-Facial function (HBGS). 2-Cure/full recovery rate.	No evidence to support the use of ES during the acute phase of recovery after FP and there is low-level evidence for patients with chronic symptoms.
Javaherian et al., 2020 [33] [SR] (4)	N = 171	LLT	No LLT treatment.	1-Facial function (HBGS). 2-Facial disability (FDI).	LLT may improve patients with sub-acute FP.
Zhang et al., 2019 [43] [SR with MA] (11)	N = 646	Acupuncture	Placebo, usual care (drugs) or any other intervention without acupuncture.	1-Cure/full recovery rate.	Acupuncture seems to be an effective therapy for FP.

SR: systematic review; ES: electrical stimulation; HBGS: House–Brackmann Grading System; FDI: facial disability index; MT: mirror therapy; SB: Sunnybrook Scale; FP: facial paralysis; LLT: low-level laser therapy; MA: meta-analysis.

**Table 2:** Primary interventions prescriptions parameters.

Primary Intervention [Reference]	Frequency (Total Sessions/Sessions Per Week)	Total Duration	Characteristics of the Treatment	Additional Treatment *
Acupuncture [42,43]	10–30/1–10	7–28 days	- Type: conventional and multi needle shallow puncture. - Size of needles: 40 mm and 30 × 45 mm. - Total number of sites: 5–13 - Length of application: 20–30 min.	None
ES [24,25]	12–157/1–21	3 weeks–1 year	- Type: monophasic, galvanic, diphasic and eutrophic. - Frequency: 2 hz–5 KHz	Drugs, hot packs, mirror therapy, facial massage and facial exercises.
LLT [33]	15–30/3–7	15–42 days	- Type: GaAs and GaAlAs. - Wavelength: 670–830 nm. - Frequency: 1000 Hz.	Facial massage and facial exercises.

ES: electrical stimulation; LLT: low-level laser therapy; GaAS: gallium–arsenide; GaAlAs: gallium–aluminum–arsenide. * Note: The additional treatment was applied in the same manner in both the experimental and control groups.

**Table 3:** Quality assessment scores (AMSTAR).

Study	1	2	3	4	5	6	7	8	9	10	11	12	13	Score
Burelo-Peregrino et al., 2020 [24]	2	0	0	2	0	2	1	2	2	0	0	1	2	14
Chen et al., 2010 [42]	2	2	2	2	2	2	2	2	2	0	0	1	0	19
Fargher and Coulson, 2017 [25]	2	2	1	2	0	2	2	2	2	2	2	2	2	23
Javaherian et al., 2020 [33]	2	0	1	2	2	2	2	2	2	0	0	0	2	17
Zhang et al., 2019 [43]	2	1	0	1	2	2	2	2	1	2	2	2	2	21

Items: 1. Explicitly described to allow replication; 2. Adequate number and range of databases; 3. Alternative searches; 4. Adequate range of keywords; 5. Non-English-language papers included in the search; 6. Inclusion criteria explicitly described to allow replication; 7. Excludes reviews that do not adequately address inclusion and exclusion criteria; 8. Two independent reviewers assessing selection bias; 9. Quality assessment explicitly described to allow replication; 10. Meta-analysis conducted on only homogeneous data or limitations to homogeneity discussed; 11. Confidence intervals/effect sizes reported where possible; 12. Conclusions supported by the meta-analysis or other data analysis findings; 13. Conclusions address levels of evidence for each intervention/comparison.

**Table 4:** Risk of bias assessment in systematic reviews through ROBIS scale.

Study	Phase 2				Phase 3
	Study Eligibility Criteria	Identification and Selection of Studies	Data Collection and Study Appraisal	Synthesis and Findings	Risk of Bias in the Review
Burelo-Peregrino et al., 2020 [24]	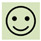	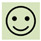	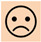	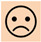	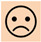
Chen et al., 2010 [42]	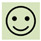	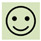	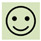	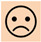	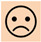
Fargher and Coulson, 2017 [25]	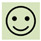	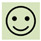	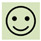	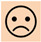	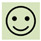
Javaherian et al., 2020 [33]	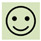	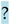	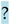	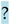	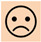
Zhang et al., 2019 [43]	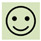	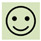	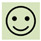	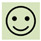	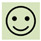

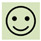
: low risk; 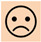: = high risk; 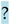: unclear risk.

## Supplementary Materials

The following supporting information can be downloaded at https://www.mdpi.com/article/10.3390/medicina61081502/s1, Supplementary Annex S1: Systematic search in several database.

## References

[1] Song A., Wu Z., Ding X., Hu Q., Di X. (2018). Neurologist Standard Classification of Facial Nerve Paralysis with Deep Neural Networks. Future Internet.

[2] Finsterer J. (2008). Management of peripheral facial nerve palsy. Eur. Arch. Oto-Rhino-Laryngol..

[3] Lassaletta L., Morales-Puebla J.M., Altuna X., Arbizu Á., Arístegui M., Batuecas Á., Cenjor C., Espinosa-Sánchez J.M., García-Iza L., García-Raya P. (2020). Parálisis facial: Guía de práctica clínica de la Sociedad Española de ORL. Acta Otorrinolaringológica Española.

[4] Mavrikakis I. (2008). Facial Nerve Palsy: Anatomy, Etiology, Evaluation, and Management. Orbit.

[5] Murakami S., Mizobuchi M., Nakashiro Y., Doi T., Hato N., Yanagihara N. (1996). Bell palsy and herpes simplex virus: Identification of viral DNA in endoneurial fluid and muscle. Ann. Intern. Med..

[6] Yetter M.F., Ogren F.P., Moore G.F., Yonkers A.J. (1990). Bell’s palsy: A facial nerve paralysis diagnosis of exclusion. Neb. Med. J..

[7] Katusic S.K., Beard C.M., Wiederholt W.C., Bergstralh E.J., Kurland L.T. (1986). Incidence, clinical features, and prognosis in Bell’s palsy, Rochester, Minnesota, 1968–1982. Ann. Neurol..

[8] Peitersen E. (2002). Bell’s palsy: The spontaneous course of 2500 peripheral facial nerve palsies of different etiologies. Acta Oto-Laryngol. Suppl..

[9] Macías-Hernández S.I., Lomelí-Rivas A., Baños T., Flores J., Sánchez M., Miranda-Duarte A. (2012). Efectos del láser de baja potencia en el tratamiento de la parálisis facial periférica aguda. Rehabilitación.

[10] Singh A., Deshmukh P. (2022). Bell’s Palsy: A Review. Cureus.

[11] Patel D.K., Levin K.H. (2015). Bell palsy: Clinical examination and management. Clevel. Clin. J. Med..

[12] Liston S.L., Kleid M.S. (1989). Histopathology of bell’s palsy. Laryngoscope.

[13] Yen T.L., Driscoll C.L.W., Lalwani A.K. (2003). Significance of House-Brackmann Facial Nerve Grading Global Score in the Setting of Differential Facial Nerve Function. Otol. Neurotol..

[14] Baude M., Guihard M., Gault-Colas C., Bénichou L., Coste A., Méningaud J.-P., Schmitz D., Natella P.-A., Audureau E., Gracies J.-M. (2023). Guided Self-rehabilitation Contract vs conventional therapy in chronic peripheral facial paresis: VISAGE, a multicenter randomized controlled trial. BMC Neurol..

[15] Díaz-Aristizabal U., Valdés-Vilches M., Fernández-Ferreras T., Calero-Muñoz E., Bienzobas-Allué E., Aguilera-Ballester L., Carnicer-Cáceres J. (2023). Efecto de la toxina botulínica tipo A en la funcionalidad, las sincinesias y la calidad de vida en secuelas de parálisis facial periférica. Neurología.

[16] Toffola E.D., Pavese C., Cecini M., Petrucci L., Ricotti S., Bejor M., Salimbeni G., Biglioli F., Klersy C. (2014). Hypoglossal-facial nerve anastomosis and rehabilitation in patients with complete facial palsy: Cohort study of 30 patients followed up for three years. Funct. Neurol..

[17] Van Swearingen J.M., Cohn J.F., Turnbull J., Mrzai T., Johnson P. (1998). Psychological Distress. Otolaryngol.—Head Neck Surg..

[18] De Almeida J.R., Guyatt G.H., Sud S., Dorion J., Hill M.D., Kolber M.R., Lea J., Reg S.L., Somogyi B.K., Westerberg B.D. (2014). Management of Bell palsy: Clinical practice guideline. Can. Med. Assoc. J..

[19] Somasundara D., Sullivan F. (2016). Management of Bell’s palsy. Aust. Prescr..

[20] Eviston T.J., Croxson G.R., Kennedy P.G.E., Hadlock T., Krishnan A.V. (2015). Bell’s palsy: Aetiology, clinical features and multidisciplinary care. J. Neurol. Neurosurg. Psychiatry.

[21] Benítez S., Danilla S., Troncoso F. (2016). Manejo Integral de la Parálisis Facial. Rev. Médica Clínica Las Condes.

[22] Weyns M., Koppen C., Tassignon M.J. (2013). Scleral Contact Lenses as an Alternative to Tarsorrhaphy for the Long-Term Management of Combined Exposure and Neurotrophic Keratopathy. Cornea.

[23] Baricich A., Cabrio C., Paggio R., Cisari C., Aluffi P. (2012). Peripheral facial nerve palsy: How effective is rehabilitation?. Otol. Neurotol..

[24] Burelo-Peregrino E.G., Salas-Magaña M., Arias-Vázquez P.I., Tovilla-Zarate C.A., Bermudez-Ocaña D.Y., López-Narváez M.L., Guzmán-Priego C.G., González-Castro T.B., Juárez-Rojop I.E. (2020). Efficacy of electrotherapy in Bell’s palsy treatment: A systematic review. J. Back Musculoskelet. Rehabil..

[25] Fargher K.A., Coulson S.E. (2017). Effectiveness of electrical stimulation for rehabilitation of facial nerve paralysis. Phys. Ther. Rev..

[26] La Touche Arbizu R., Escalante K., Linares M.T., Mesa J. (2008). Efectividad del tratamiento de fisioterapia en la parálisis facial periférica. Revisión sistemática. Rev. Neurol..

[27] Nakano H., Fujiwara T., Tsujimoto Y., Morishima N., Kasahara T., Ameya M., Tachibana K., Sanada S., Toufukuji S., Hato N. (2024). Physical therapy for peripheral facial palsy: A systematic review and meta-analysis. Auris Nasus Larynx.

[28] Cardoso J.R., Teixeira E.C., Moreira M.D., Fávero F.M., Fontes S.V., de Oliveira A.S.B. (2008). Effects of exercises on Bell’s palsy: Systematic review of randomized controlled trials. Otol. Neurotol..

[29] Pourmomeny A.A., Asadi S. (2014). Management of synkinesis and asymmetry in facial nerve palsy: A review article. Iran. J. Otorhinolaryngol..

[30] Teixeira L.J., Soares B.G.D.O., Vieira V.P., Prado G.F. (2008). Physical therapy for Bell’s palsy (idiopathic facial paralysis). Cochrane Database of Systematic Reviews.

[31] Vaughan A., Gardner D., Miles A., Copley A., Wenke R., Coulson S. (2020). A systematic review of physical rehabilitation of facial palsy. Front. Neurol..

[32] Granero-Pérez M., Martí-Amela A.B. (2021). Physiotherapy in idiopathic facial paralysis. A systematic review. Fisioterapia.

[33] Javaherian M., Attarbashi Moghaddam B., Bashardoust Tajali S., Dabbaghipour N. (2020). Efficacy of low-level laser therapy on management of Bell’s palsy: A systematic review. Lasers Med. Sci..

[34] Castaldo M., Sellitto G., Ruotolo I., Berardi A., Galeoto G. (2024). The Use of Mirror Therapy in Peripheral Seventh Nerve Palsy: A Systematic Review. Brain Sci..

[35] Alakram P., Puckree T. (2011). Effects of electrical stimulation in early Bells palsy on facial disability index scores. South Afr. J. Physiother..

[36] Shamseer L., Moher D., Clarke M., Ghersi D., Liberati A., Petticrew M., Shekelle P., Stewart L.A., PRISMA-P Group (2015). Preferred reporting items for systematic review and meta-analysis protocols (PRISMA-P) 2015: Elaboration and explanation. BMJ.

[37] Booth A., Clarke M., Ghersi D., Moher D., Petticrew M., Stewart L. (2011). An international registry of systematic-review protocols. Lancet.

[38] Bougioukas K.I., Liakos A., Tsapas A., Ntzani E., Haidich A.B. (2018). Preferred reporting items for overviews of systematic reviews including harms checklist: A pilot tool to be used for balanced reporting of benefits and harms. J. Clin. Epidemiol..

[39] Barton C.J., Webster K.E., Menz H.B. (2008). Evaluation of the scope and quality of systematic reviews on nonpharmacological conservative treatment for patellofemoral pain syndrome. J. Orthop. Sports Phys. Ther..

[40] Whiting P., Savović J., Higgins J.P., Caldwell D.M., Reeves B.C., Shea B., Davies P., Kleijnen J., Churchill R., ROBIS group (2016). ROBIS: A new tool to assess risk of bias in systematic reviews was developed. J. Clin. Epidemiol..

[41] Fuentes-Aparicio L., Cuenca-Martínez F., Muñoz-Gómez E., Mollà-Casanova S., Aguilar-Rodríguez M., Sempere-Rubio N. (2023). Effects of therapeutic exercise in primary dysmenorrhea: An umbrella and mapping review. Pain Med..

[42] Chen N., Zhou M., He L., Zhou D., Li N., He L. (2010). Acupuncture for Bell’s palsy. Cochrane Database of Systematic Reviews.

[43] Zhang R., Wu T., Wang R., Wang D., Liu Q. (2019). Compare the efficacy of acupuncture with drugs in the treatment of Bell’s palsy. Medicine.

[44] VanSwearingen J.M., Brach J.S. (1996). The Facial Disability Index: Reliability and validity of a disability assessment instrument for disorders of the facial neuromuscular system. Phys. Ther..

[45] House J.W., Brackmann D.E. (1985). Facial nerve grading system. Otolaryngol. Head Neck Surg..

[46] Di Pietro A., Cameron M., Campana V., Leyes L., Cinat J.A.I.Z., Lochala C., Johnson C.Z., Hilldebrand A., Loyo M. (2023). Efficacy of adding selective electrical muscle stimulation to usual physical therapy for Bell’s palsy: Immediate and six-month outcomes. Eur. J. Transl. Myol..

[47] Fahmy S.M., Ahmed H.F., Alhirsan S.M., Bahey El-Deen H.A., Ameer M.A. (2025). Effect of acupuncture combined with moxibustion therapy on the recovery rate of Bell’s palsy: A double-blind randomized control study. J. Bodyw. Mov. Ther..

[48] Li X.-W., Chen J.-J., Shu Y.-L., Deng X.-Y., Zhang Y., Yang J., Shi H.-P. (2025). Effects of acupuncture on Bell’s palsy patients in the acute stage based on the surface electromyography. Acupunct. Res..

[49] Martín Piñero B., Pérez Rodríguez E., Yumar Carralero A.C., Henández Calzadilla M.d.l.Á., Lamarque Martínez V.H., Castillo Bueno E. (2017). Efectividad de la rehabilitación en la parálisis de Bell. Rev. Cuba. Med. Física Rehabil..

[50] Kandakurti P.K., Shanmugam S., Basha S.A., Amaravadi S.K., Suganthirababu P., Gopal K., George G.S. (2020). The effectiveness of low-level laser therapy combined with facial expression exercises in patients with moderate-to-severe Bell’s palsy: A study protocol for a randomised controlled trial. Int. J. Surg. Protoc..

[51] Johannes F., Pekacka-Egli A.M., Köhler S., Disko A., von Meyenburg J., Bujan B. (2025). EMG-Triggered Functional Electrical Stimulation for Central Facial Palsy Following Stroke: A Clinical Case Report. Brain Sci..

[52] Tuncay F., Borman P., Taşer B., Ünlü İ., Samim E. (2015). Role of Electrical Stimulation Added to Conventional Therapy in Patients with Idiopathic Facial (Bell) Palsy. Am. J. Phys. Med. Rehabil..

[53] Lin H.W., Chen H.C., Lin L.F., Tam K.W., Kuan Y.C. (2024). Laser therapy for Bell’s palsy: A systematic review and meta-analysis of randomized trials. Lasers Med. Sci..

[54] Alayat M.S.M., Elsodany A.M., El Fiky A.R. (2014). Efficacy of high and low level laser therapy in the treatment of Bell’s palsy: A randomized double-blind placebo-controlled trial. Lasers Med. Sci..

