# Supplementary material for: Effectiveness of Non-Pharmacological Interventions in Patients with Facial Paralysis: An Umbrella and Mapping Review
Source: Medicina (Kaunas). 2025 Aug 21;61(8):1502. doi: 10.3390/medicina61081502 (PMC12388444; doi:10.3390/medicina61081502)
Supplement: Supplementary file 1 [file medicina-61-01502-s001.zip › medicina-3787568-supplementary.pdf]

Annex S1. Systematic search in several database

PubMed

("bell palsy"[MeSH Terms] OR ("bell"[All Fields] AND "palsy"[All Fields]) OR "bell palsy"[All Fields] OR ("bell s"[All Fields] AND "palsy"[All Fields]) OR "bell s palsy"[All Fields] OR ("facial paralysis"[MeSH Terms] OR ("facial"[All Fields] AND "paralysis"[All Fields]) OR "facial paralysis"[All Fields]) OR (("facial nerve"[MeSH Terms] OR ("facial"[All Fields] AND "nerve"[All Fields]) OR "facial nerve"[All Fields]) AND ("paresis"[MeSH Terms] OR "paresis"[All Fields] OR "pareses"[All Fields])) OR (("peripheral"[All Fields] OR "peripherally"[All Fields] OR "peripherals"[All Fields] OR "periphereal"[All Fields] OR "peripheric"[All Fields] OR "periphericall y"[All Fields]) AND ("facial paralysis"[MeSH Terms] OR ("facial"[All Fields] AND "paralysis"[All Fields]) OR "facial paralysis"[All Fields] OR ("facial"[All Fields] AND "palsy"[All Fields]) OR "facial palsy"[All Fields])) OR ("facial paralysis"[MeSH Terms] OR ("facial"[All Fields] AND "paralysis"[All Fields]) OR "facial paralysis"[All Fields] OR ("peripheral"[All Fields] AND "facial"[All Fields] AND "paralysis"[All Fields]) OR "peripheral facial paralysis"[All Fields])) AND ("physical therapy modalities"[MeSH Terms] OR ("physical"[All Fields] AND "therapy"[All Fields] AND "modalities"[All Fields]) OR "physical therapy modalities"[All Fields] OR "physiotherapies"[All Fields] OR "physiotherapy"[All Fields] OR ("physical

therapy modalities"[MeSH Terms] OR ("physical"[All Fields] AND "therapy"[All Fields] AND "modalities"[All Fields]) OR "physical therapy modalities"[All Fields] OR ("physical"[All Fields] AND "therapy"[All Fields]) OR "physical therapy"[All Fields])) AND "systematic review"[Title/Abstract]

PubMed

("bell palsy"[MeSH Terms] OR ("bell"[All Fields] AND "palsy"[All Fields]) OR "bell palsy"[All Fields] OR ("bell s"[All Fields] AND "palsy"[All Fields]) OR "bell s palsy"[All Fields] OR ("facial paralysis"[MeSH Terms] OR ("facial"[All Fields] AND "paralysis"[All Fields]) OR "facial paralysis"[All Fields]) OR (("facial nerve"[MeSH Terms] OR ("facial"[All Fields] AND "nerve"[All Fields]) OR "facial nerve"[All Fields]) AND ("paresis"[MeSH Terms] OR "paresis"[All Fields] OR "pareses"[All Fields])) OR (("peripheral"[All Fields] OR "peripherally"[All Fields] OR "peripherals"[All Fields] OR "periphereal"[All Fields] OR "peripheric"[All Fields] OR "peripherically"[All Fields]) AND ("facial paralysis"[MeSH Terms] OR ("facial"[All Fields] AND "paralysis"[All Fields]) OR "facial paralysis"[All Fields] OR ("facial"[All Fields] AND "palsy"[All Fields]) OR "facial palsy"[All Fields])) OR ("facial paralysis"[MeSH Terms] OR ("facial"[All Fields] AND "paralysis"[All Fields]) OR "facial paralysis"[All Fields] OR ("peripheral"[All Fields] AND "facial"[All Fields] AND "paralysis"[All Fields]) OR "peripheral facial paralysis"[All Fields])) AND ("exercise"[MeSH

Terms] OR "exercise"[All Fields] OR "exercises"[All Fields] OR "exercise therapy"[MeSH Terms] OR ("exercise"[All Fields] AND "therapy"[All Fields]) OR "exercise therapy"[All Fields] OR "exercising"[All Fields] OR "exercise s"[All Fields] OR "exercised"[All Fields] OR "exerciser"[All Fields] OR "exercisers"[All Fields] OR (("face"[MeSH Terms] OR "face"[All Fields] OR "facial"[All Fields] OR "facials"[All Fields]) AND ("exercise"[MeSH Terms] OR "exercise"[All Fields] OR "exercises"[All Fields] OR "exercise therapy"[MeSH Terms] OR ("exercise"[All Fields] AND "therapy"[All Fields]) OR "exercise therapy"[All Fields] OR "exercising"[All Fields] OR "exercise s"[All Fields] OR "exercised"[All Fields] OR "exerciser"[All Fields] OR "exercisers"[All Fields]))) AND "systematic review"[Title]

EMBASE

('facial nerve paralysis'/exp OR '7th cranial nerve palsy' OR '7th cranial nerve paralysis' OR '7th cranial nerve paresis' OR 'central facial nerve palsy' OR 'cranial nerve paralysis, 7th' OR 'facial diplegia' OR 'facial nerve palsy' OR 'facial nerve paralysis' OR 'facial nerve paresis' OR 'facial palsy' OR 'facial paralysis' OR 'facial paresis' OR 'facialis paralysis' OR 'paralysis, facial nerve' OR 'prosopoplegia' OR 'recurrent facial nerve palsy' OR 'seventh cranial nerve palsy' OR 'seventh cranial nerve paralysis' OR 'seventh cranial nerve paresis' OR 'bell palsy'/exp OR 'bell palsy' OR 'bell paralysis' OR 'bell`s palsy' OR 'bell`s paralysis' OR 'bells palsy' OR

'acute idiopathic facial neuropathy' OR 'acute inflammatory facial neuropathy' OR 'facial paralysis, idiopathic' OR 'herpetic facial paralysis' OR 'idiopathic acute facial neuropathy' OR 'idiopathic facial palsy' OR 'idiopathic facial paralysis') AND ('physiotherapy'/exp OR 'physical therapy' OR 'physical therapy (speciality)' OR 'physical therapy (specialty)' OR 'physical therapy modalities' OR 'physical therapy service' OR 'physical therapy speciality' OR 'physical therapy specialty' OR 'physical therapy techniques' OR 'physical treatment' OR 'physio therapy' OR 'physiotherapy' OR 'physiotherapy department' OR 'therapy, physical' OR 'exercise'/exp OR 'biometric exercise' OR 'effort' OR 'exercise' OR 'exercise capacity' OR 'exercise performance' OR 'exercise training' OR 'exertion' OR 'fitness training' OR 'fitness workout' OR 'physical conditioning, human' OR 'physical effort' OR 'physical exercise' OR 'physical exertion' OR 'physical work-out' OR 'physical workout') AND ('systematic review'/exp OR 'review, systematic' OR 'systematic review')

Scopus

(TITLE-ABS-KEY("peripheral facial paralysis" OR "facial palsy" OR "Bell's palsy" OR "Bell's paralysis" OR "peripheral facial palsy")) AND (TITLE-ABS-KEY-AUTH("physiotherapy" OR "exercise" OR "physical therapy" OR "facial exercises" OR "electrotherapy")) AND (TITLE-ABS-KEY("Systematic review" OR "Meta-analysis"))

CINAHL Complete (through University of Valencia)

peripheral facial paralysis OR (facial palsy or hemifacial paralysis or bell palsy) AND (physiotherapy or physical therapy or rehabilitation) OR (exercise or physical fitness or physical activity) AND (systematic reviews or meta analysis or meta- analysis)
